# Editorial Department

**Published:** 1866-10

**Authors:** 


### Editorial Department.

---

What nature does in disease, and what the physician can do, embraces the whole practical field of medicine, and cannot be expressed in detail, or outlined even, without including what we know, and what we do not know. Any just appreciation of what nature does, grows out of a clear understanding of the natural history of disease, of its symptoms, causes and termination, uninfluenced by remedies. A clear discrimination between the workings of natural causes and the influences of art, are necessary in arriving at truthful estimates of the value of medicine. There is no end to the amount of published discovery in our profession, and great sagacity is required, to rightly estimate the claims which are made to improvement in every department. Great changes and wonderful discoveries have actually been made within the last few years—the old routine of thought has been broken in upon; authority, another term for the influence of great names, has lost some of its control—physicians follow less dependently the guidance of distinguished leadership, are more at liberty to observe for themselves, and less ready to accept the teachings of others without personal investigation. The effect of this condition of things, is manifest in the advance of scientific research, in the rejection of errors which had been entertained for years, and in a more rational appreciation of the influence of natural causes in recovery from disease or its termination in death.

Disease may be said to be, in general terms, deviations from health in function or structure—accidents which occur in the growth, formation and renewal, of animal life. We are born, grow old and die, death itself as truly in the natural course, as life and growth. Disorders of function, interruption in the smooth working of mortal machinery constitute the major part of the ills of life, while deviations in structure or growth, comprise the more intractable maladies. The forces by which animal life is propelled are sufficiently in excess of the demand required for ordinary locomotion, so that the "up grade" of disease is generally, almost always, for a time at least, overcome, until at length, when time has lessened the moving force, decay and death take the places of life and growth; then diseases cease to disappear, and the normal affinities of life, give place to the precipitations and dissolutions of death. Disease of function manifests natural tendency to recovery, and in general ends in it, when uninfluenced by art; disease of structure has tendency in this dfrection less strongly marked, and that form of it which has been called malignant appears wholly incorrigible, neither nature or art having any controlling influence. These truths have been ignored to a certain extent, and even now physicians unwillingly admit the dependence they place upon the operation of natural causes It has been made to appear if possible that results were wholly due to medicine which were really due to nature alone, as though the credit of cure, was greater than the honor of perfect knowledge, of complete familiarity with the workings of nature. The true physician is now more widely and manifestly separated from the empiric, by a full recognition of these facts, than by any differences in the use of remedies. The more a physician knows of what nature does in disease and what a physician can do, the greater his confidence in it, and the less his dependence upon drugs.

The great tendency of our time, is to attempt too much; every improvement is heralded with the enthusiasm of its discoverer; every new operation or modification of an old one is placed pre-eminently before the medical public; even the trivial detail of unimportant manipulation comes announced as something new, until the ranks of the profession are filled with presumption and pretention scarceley less than the lines of unscrupulous charlatans and mountebanks.—The public have no appreciation at all of what nature does in disease, and half believe that the physician holds the governing control. What medicine can do, has always been written and talked, until the most senseless propositions are easily received as established truths. Failure upon the part of physicians to impress the public with this great fundamental and central truth, has no doubt contributed to the prevalence of error, and a desire with invalids to change their line of practice whenever a more *promising* plan is presented, regardless of every consideration, except, that they are not better, believing that the medicine must be in fault, and not the nature of disease. The united influence of the educated medical mind upon the community is all powerful; and when it is faithfully directed to the impression of an essential truth, it cannot fail of obtaining the desired object. The real truth in medicine is but rarely and imperfectly presented to the public, so that from necessity they are disqualified to judge of its merits. It has never been attempted to educate the masses in matters of disease, and greater ignorance prevails in this direction, with in other respects intelligent individuals, than in any other department of knowledge. Diseases and the influence of remedies, are not made the subjects of popular discussion by intelligent physicians, but the whole field is given over to the ignorant, unscrupulous pretenders, who flood the world with the most monstrous absurdities, for personal gain, and practice upon the credulous and superstitious the vilest impositions.

It will be replied, that the great mass of mankind are uneducated, and consequently incapable of impression in this direction, and that it is unbecoming the dignity of the profession to attempt to give the public its principles of action. Too long this objection has been the controling argument, until it appears probable that the best and only remaining means of retaining confidence and insuring respect, is by plain and truthful declaration of what nature does in disease, and what the physician can do. It is true that the intelligent in other departments of knowledge, sometimes adopt the most foolish and inconsistent notions in medicine, but this grows out of the truth, that they have never been instructed in what nature or medicine can do, and though wise in some respects, they yet know nothing at all in others. It is painful to observe the ignorance which often prevails with members of the other learned professions, in all the essential truths of medicine; to see the Doctor of Divinity advocating inconsistencies in medicine, greater than idol worship, in theology. Legal minds appear to have digested the force of evidence more perfectly, and to their credit it may be said, they are less liable to adopt palpable error; but it is not improbable but even with the legal profession some false premises are assumed unknowingly. Of these conditions no complaint can reasonably be made; physicians even "wander upon the dark mountains and perish." Men are not capable of seeing or judging alike, and there is no hope of an approaching day, "when truth shall be seen unmixed with error. While this is true, it is not right, it is not fair, it is not being true to ourselves, the profession, or the world, if the most earnest effort, both private and public, is not made, to prevent the adoption of error, to place the truth fairly by its side, and to give appreciating people opportunity to judge correctly. The popular press is given over wholly to the dominion of quackery; its advertisement sheets and editorial columns are alike filled with copy written by the charlatans and impostors, who throng our cities and large towns, and announce their "distinguished names and great cures." We would not wrest from their unholy hands these powerful weapons, but would rather protect their innocent victims. We would allow the itinerant mountebank to purchase newspaper puffs while his funds last, but would cut off his supplies by infusing into the community a true knowledge of what nature does and what a physician can do.

